# Supplementary material for: Birth weight, growth, nutritional status and mortality of infants from Lambaréné and Fougamou in Gabon in their first year of life
Source: PLoS One. 2021 Feb 9;16(2):e0246694. doi: 10.1371/journal.pone.0246694 (PMC7872243; doi:10.1371/journal.pone.0246694)
Supplement: S1 Table — (DOCX) [file pone.0246694.s001.docx]

**S1 Table. Sensitivity analysis.**

|  | | Baseline | |  | Month 1 | | *p-*value | Month 9 | | *p*value | Month 12 | | *p-*Value |
| --- | --- | --- | --- | --- | --- | --- | --- | --- | --- | --- | --- | --- | --- |
|  |  | n | % |  | n | % |  | n | % |  | n | % |  |
| N | | 907 | 100 |  | 765 | 84.3 |  | 575 | 63.4 |  | 576 | 63.5 |  |
| Place of birth | |  |  |  |  |  |  |  |  |  |  |  |  |
|  | Maternity | 827 | 91 |  | 694 | 83.9 | 0.4 | 520 | 62.9 | 0.3 | 522 | 63.1 | 0.4 |
|  | Home | 80 | 9 |  | 70 | 87.5 |  | 55 | 68.8 |  | 54 | 67.5 |  |
| Mean gestational age (weeks) | | 41 |  |  | 41 |  |  | 41 |  |  |  |  |  |
| Mean birth Weight (g) | |  |  |  |  |  |  |  |  |  |  |  |  |
|  | Overall population | 2994 | - |  | 2998 | - |  | 3032 | - |  | 3010 | - |  |
|  | LBW | 2172 | - |  | 2181 | - |  | 2279 | - |  | 2242 | - |  |
|  | NBW | 3113 | - |  | 3118 | - |  | 3119 | - |  | 3111 | - |  |
| LBW | | 115 | 12.7 |  | 91 | 79 | 0.1 | 60 | 52.2 | 0.008 | 67 | 58.3 | 0.2 |
| NBW | | 792 | 87.3 |  | 674 | 85 |  | 515 | 65 |  | 509 | 64.3 |  |
| Preterm* | |  |  |  |  |  |  |  |  |  |  |  |  |
|  | Yes | 50 | 5.7 |  | 43 | 86 | 0.8 | 28 | 56 | 0.2 | 33 | 66 | 0.9 |
|  | No | 832 | 94.3 |  | 705 | 84.7 |  | 545 | 65.5 |  | 542 | 65.1 |  |
| Infant Sex** | |  |  |  |  |  |  |  |  |  |  |  |  |
|  | Male | 454 | 50.7 |  | 397 | 87.4 | 0.05 | 285 | 62.8 | 0.3 | 285 | 87.4 | 0.4 |
|  | Female | 442 | 49.3 |  | 366 | 82.8 |  | 289 | 65.6 |  | 289 | 82.8 |  |
| Mother Literacy | |  |  |  |  |  |  |  |  |  |  |  |  |
|  | Literate | 745 | 82.1 |  | 624 | 83.8 | 0.4 | 467 | 62.7 | 0.3 | 462 | 62 | 0.04 |
|  | Illiterate | 162 | 17.9 |  | 140 | 86.4 |  | 108 | 66.7 |  | 114 | 70.4 |  |
| Maternal age (year) | |  |  |  |  |  |  |  |  |  |  |  |  |
|  | **<20** | 287 | 31.6 |  | 247 | 86.1 | 0.2 | 174 | 60.6 | 0.05 | 174 | 60.6 | 0.03 |
|  | **20-35** | 549 | 60.6 |  | 454 | 82.7 |  | 347 | 63.2 |  | 347 | 63.2 |  |
|  | **>35** | 71 | 7.8 |  | 63 | 88.7 |  | 54 | 76.1 |  | 55 | 77.5 |  |
| Congenitale Abnormalities*** | | |  |  |  |  |  |  |  |  |  |  |  |
|  | Present | 22 | 2.4 |  | 17 | 77.3 | 0.3 | 14 | 63.6 | 0.9 | 11 | 50 | 0.1 |
|  | Absent | 879 | 97.6 |  | 742 | 84.4 |  | 561 | 63.8 |  | 565 | 64.3 |  |

* 25 missing data, ; ** 1 missing data ; ***6 missing data
