# Supplementary material for: Birth weight, growth, nutritional status and mortality of infants from Lambaréné and Fougamou in Gabon in their first year of life
Source: PLoS One. 2021 Feb 9;16(2):e0246694. doi: 10.1371/journal.pone.0246694 (PMC7872243; doi:10.1371/journal.pone.0246694)
Supplement: Attachment — Submitted filename: Response to Reviewers-final.docx [file pone.0246694.s002.docx]

**Point by point responses to reviewer's Questions**

**Reviewer #1:**

This manuscript is on an important topic, but the writing is somewhat confusing and needs a close grammar proofread; not all of the numbers in the text match the tables; and I have certain concerns about the data and analyses as presented.

**Reply**: **We thank the reviewer for the thorough assessment of our manuscript and the queries raised. This has tremendously been helpful in improving the quality of the manuscript. Please, we have performed a grammar proofread and corrected the mismatches in the tables.**

**Major concerns:**

My 2 major concerns are about the amount of missing data and the selected confounders. The most concerning issue for me is that loss to follow up is a huge limitation. The study lost either 25 or about 45% of the sample, depending on how you choose the denominator (one month vs. baseline). This issue is not addressed anywhere in the limitations or in the manuscript. I think the authors need to present some sensitivity analyses and say something about how they differed than the study participants who were retained, and how those differences might influence the study's findings.

**Reply:** **This is correct that there were a lot of losses to follow-up from 9 months that can be explained by the high mobility of the population in Gabon. This limitation has been addressed by conducting sensitivity analyses as advised by the reviewer. The findings of the sensitivity analyses are shown in the supplementary table added. Mention of the sensitivity analyses have been added in the methods, results and discussion sections.**

It's also not clear which children are in the KM and hazard analyses--have the authors censored the LTFs at last seen date, or just ignored them in that analysis?

**Reply: 907 infants were included in the KM hazard analysis. LTFs were included in the analysis and censored at the last contact date.**

My other major concern is that the authors have some specific confounders they controlled for, but why these specific ones were chosen are mysterious. There are other factors also possibly relevant--breast feeding history, complementary feeding history, family wealth, number of other kids in household, availability of & use of antenatal care--so what was the rationale for including these specific confounders and nothing else?

**Reply:** **Some factors including mother’s age and literacy, infant’s sex and prematurity, breast feeding history, complementary feeding history, family wealth, number of other kids in household, availability of & use of antenatal care are recognised as risk factors for malnutrition. The main study was conducted to evaluate intermittent preventive treatment of malaria during pregnancy, for which some of those variables were recorded and used in our analysis. The main study was designed as a randomized clinical trial with the assumption that confounders would be evenly distributed between the treatment arms, they were therefore not systematically recorded. We have mentioned that limitation in the manuscript (line 306-307). It can be believed that the socioeconomic factors have minimal effect as in the context of the clinical trial as standard care was provided to all participants and their offspring.**

**Minor concerns**

Line 68-71: this sentence is hard to understand. I'd break it into 2 because you seem to have 2 concepts here.

**Reply:** **Sentence was broken into two sentences as advised**

Line 80: "Once established, stunting and its effects typically become permanent. Stunting is an appropriate indicator for chronic malnutrition"...There's some controversy about this. Some studies have shown trajectories in which children recover and others in which they don't. Early intervention is believed to help with this. You need some citations for your contention here.

**Reply:** **Thank you for this suggestion. We added a citation (Dewey and Begum, 2011) to support the statement**

Line 82:"Underweight is most commonly used as nutritional indicator due to difficulties..." This statement again needs some citation. DHS, MICS, and a lot of other national programs use stunting and wasting. Saying that underweight is most commonly used needs to be backed up with some evidence.

**Reply:** **We added citation (Prendergast and Humphrey, 2014)** **to support the statement.**

Line 133: "For those with no weight available at birth but in the first week of life in case of home deliveries weight was estimated using a regression model". This needs some explanation. How do you estimate weight with regression?

**Action: There is a linear regression model described by Greenwood and colleagues (1992) adjusting the birthweight measured after the day of birth. On average, infants’ weight 6.3 grams less each day after birth. In the analysis the weight at birth was estimated using this coefficient for those children weighed after day 0. The fitted values by the linear model are very similar to the values estimated by Lowess regression.**

**Below, the estimations from the main trial as published in Plos Medicine (Gonzales et al. 2014)**

Table 1: Preterm birth totals don't add to 907 or 115. I suggest that the authors present "n"s when they don't match the stated denominator.

**Reply:** **The reviewer is correct on that point, we have corrected the table with “n”s for each variable and since the total population at baseline was 907, we have indicated with “*” the variables with missing data.**

Line 189: Authors haven't defined a weight gain ratio.

**Reply:** **This is correct, it has now been defined in the text. Weight gain ratio is the ratio of the weight of infants at the visit divided by the weight of the infants at the previous visit. It has been added in the manuscript line 155-157.**

Line 200: if the authors are presenting decimal places in the table, it makes sense to match that in the text.

**Reply:** **We rounded in order to make the result easier to read but reviewer’s comment is relevant so we have shown the decimal places in the text as well.**

Line 208: "There were more significantly observed underweight LBW infants compared to their normal weight counterpart". I did not understand the sentence. what does it mean to be significantly observed underweight?

**Reply:** **The sentence was reformulated. “There were significantly more underweight observed among infants born with low weight compared to their normal birth weight counterpart” (line 217-219)**

Lines 200-212: I found it challenging to read and follow this paragraph. Maybe the authors could restructure this so that they talk about each endpoint separately? So, first stunting, and what's associated. Then wasting, and what's associated with that, etc.

**Reply:** **Text was restructured**

Lines 221-222: These numbers should all match what is in table 4. I found this paragraph also very confusing. LBW was associated with higher stunting at months 1 AND month 12, but not in between. These are all one model, correct? So preterm birth is associated with stunting independent of LBW?

**Reply:** **Numbers were corrected in the text to match what is in table 4. That’s the same model, and it showed actually stronger evidence of an association of LBW and stunting at month 1 and month 12. For month 9, there was obviously a trend of an association too as the estimates of OR was 2.0, however the 96%CI crossed 0. That weak evidence at 9 months is not yet fully understood but we think that it reflects what is shown in Figure 3 as there is an important weight gain before 9 months.**

Discussion: the English here needs some boosting also.

**Reply:** **The English was reviewed and the section was rewritten to address the language issue**

Lines 263-265: I didn't understand what it means to have a nutritional gain if the prevalence of stunting and underweight increased and prevalence of wasting stayed the same. Perhaps reword this? Do you mean that mean weight increased for all children?

**Reply:** **This was actually a mistake that has been corrected, thank you. We meant weight gain instead.**

Lines 272-274: "We hypothesize that this catching up in weight gain is at least in part due to the particular attention caused by mothers and the health care system allowing children born with LBW to acquire a normal weight" What is this extra attention? Can you be more specific about Gabon's practices in the health care system to address these nutritional challenges in LBW children?

**Reply:** **We mean that the important weight gain observed in the LBW infants’ group may be explained by the particular attention given to that group and the subsequent restoration of growth that restricted in utero.**

Lines 293-296: I don’t understand this paragraph. LBW is an independently associated with stunting at 12 months in this setting--but what does that have to do with weight deficit since birth? These 2 things are probably true, but the authors haven't really connected them coherently here.

**Reply:** **The sentence was rephrased to highlight the potential mechanisms of LBW involved, see action above.**

Lines 303-304: "This indicates that 302 health interventions need to focus primarily on the two first months of life, particularly for newborns with LBW." I'm not sure this is a reasonable suggestion. What do the authors mean by "primarily"? Even among those who survive, a high proportion have poor nutrition outcomes. So, I'm not sure it makes sense to say that that health care programs should put most focus on the first 2 months. Obviously, something needs to be done but the children who survive also need health care support. Also, the authors don’t mention what proportion of deaths occur as neonates. Health interventions often don't help to move the needle on neonatal death. What might help with these early deaths is antenatal care, facility delivery in a well-stocked facility, etc. Other health service interventions may be of more benefit in children after the first few weeks of life.

**Reply:** **We have reformulated our recommendations and it is indeed better to promote interventions targeting pregnant women to prevent babies to be born too small.**

**Reviewer #2:**

The manuscript presented an interesting data on the significance of LWB for survival and nutritional status of infants in Gabon. While the findings of the analysis are not new, they may help to consolidate the existing knowledge on the topic. In general, the manuscript is also well written. I recommend the authors accommodate the following comments:

-Exhaustive list of potential confounders along with approach used for selecting the variables for multivariable adjustment should be provided. For example, it is not clear how the authors decided to adjust the multivariable models for infant sex, maternal age and maternal literacy.

**Reply:** **Some factors including mother’s age and literacy, infant’s sex and prematurity, breast feeding history, complementary feeding history, family wealth, number of other kids in household, availability of & use of antenatal care are recognised as risk factors for malnutrition. The main study was conducted to evaluate intermittent preventive treatment of malaria during pregnancy, for which some of those variables were recorded and used in our analysis. The main study was designed as a randomized clinical trial with the assumption that confounders would be evenly distributed between the treatment arms, they were therefore not systematically recorded. We have mentioned that limitation in the manuscript (line 306-307). It can be believed that the socioeconomic factors have minimal effect as in the context of the clinical trial as standard care was provided to all participants and their offspring.**

-The adequacy of the sample size for comparing mortality between LBW and normal birthweight infants should be evaluated through post-hoc power calculation.

**Reply:** **we calculated post-hoc power for the comparison of mortality between LBW and normal birth weight and we obtained 95 %**.

-The possibility of loss to follow up bias should be explored by comparing the characteristics of the study participants retained and dropped out of the study.

**Reply:** **This is correct, we have now addressed that limitation by conducting sensitivity analyses that showed that the baseline characteristics of the retained participants were similar to that of the whole population including the lost to follow up. This is reported in the supplementary table.**

-The basic characteristics of exposed and non-exposed subjects should be described and statistically compared at the beginning of the Results section.

**Reply:** **We included exposed and non-exposed subjects in baseline characteristics table.**
