# Supplementary material for: Birth weight, growth, nutritional status and mortality of infants from Lambaréné and Fougamou in Gabon in their first year of life
Source: PLoS One. 2021 Feb 9;16(2):e0246694. doi: 10.1371/journal.pone.0246694 (PMC7872243; doi:10.1371/journal.pone.0246694)
Supplement: Attachment — Submitted filename: Rebuttal point-by-point response to the reviewers.docx [file pone.0246694.s003.docx]

**Rebuttal point-by-point response tot he reviewers**

**Review Comments to the Author**

Please use the space provided to explain your answers to the questions above. You may also include additional comments for the author, including concerns about dual publication, research ethics, or publication ethics. (Please upload your review as an attachment if it exceeds 20,000 characters)

Reviewer #1: I thank the authors for their efforts to address the comments that the other reviewer and I made. I appreciate their time. However, I still have one large concern and a few minor changes and suggestions.

MAIN large reservation:
I don’t think that the authors entirely understood my request to do a sensitivity analysis of those who were lost to follow up, or if they did, it’s not reflected in the supplemental table. In lines 317-323 “sensitivity analyses that demonstrated that the baseline characteristics of the retained children were not different from the entire population at baseline [**assuming that those dropped out were similar to those retained with regard to these characteristics**].” I have bracketed the concerning portion because in my view, that assumption renders the authors’ sensitivity analysis invalid.

What I am thinking of here is a sensitivity analysis in its simplest form as follows: those who were LTFU are one category and those who were never LTFU or missing are another. Then compare those 2 categories on things like LBW, malnutrition, preterm birth, infant sex, etc., What I see in the Supplementary table are descriptions of those who were retained at different time points, with no p values or comparisons. Just based on the numbers I see in the table, at 9 mo, the authors have 60/115 LBW kids (~52%) vs 515/792 normal birthweight kids (~65%). Which could very well be a statistically significant difference, but we don't know. The mean birthweight in your LBW children at 12 months was somewhat higher than in the LBW children at baseline. My point is that the children that were lost may be different in meaningful ways than the ones who were retained. My suggestion is to figure out whether there were differences, present p values in the supplement table to reassure the readers, and if there are differences, mention in the discussion whether you think they’d impact the results and how. It may be that you suspect that if everyone were there, you’d get even stronger associations.
**Reply: We thank the reviewer for the very relevant comment and we agree with the concerns and we also believe that it is an added information that better helps interpret the findings. We have added some more details in the supplementary table like the percentages per line of those present at any specific time point compared to baseline. a statistical test was performed and the P values obtained have been added in the table. At 9 months, there are significantly more infants from the LBW group that did not attend that visit compared to the NBW group.**

Minor comments and proofreading:
Line 35, don’t need comma after “both”

**Reply: correction made, thanks.**
Line 40 : included between 2009 and 2012 makes it sound like these are “n”s . Suggest you move the word “included” to after 2012.

**Reply: the suggestion has been taken into account and the move made. the sentence now reads: “...conducted between 2009 and 2012, included infants…”**

Line 48: It could be the same number of infants that were LBW, it’s just that you’ve lost more during follow up. What are the actual ns? Is it same kids?

**Reply: Following the sensitive analysis, it appears that a significant number of LBW kids was missing at 9 months visit compared to the other group. The proportions remain similar at all the other time-points between LBW group and NBW group. This is now mentioned in the discussion.**

Line 55: ratio, not ration.

**Reply: typo was corrected**


Body:
line 77 “”stunting is reported to be a strong marker of healthy growth”. It’s more like unhealthy growth, no?
**Reply: that is true, typo was corrected**

line 86” While stunting wasting and underweight…low birth weight…” maybe make that the first sentence of the LBW paragraph
**Reply: Done**
line 139: “like describe elsewhere”. This is not great grammar and not especially useful. I’d suggest you say something like “calculated using standard formula” and cite maybe DHS statistics or WHO web page that talks about how to calculate,
**Reply: Sentence was reworded**
line 139
Height-for-age Z-score (HAZ), weight-for-age Z-score (WAZ) and weight-for-height Z-score (WHZ) were used to define respectively stunting, underweight and wasting. HAZ <-2 standard deviations (SD), WAZ <-2 141SD and WHZ <-2 SD.

Suggest you reword to make complete sentences:
Ex: Height-for-age Z-score (HAZ, <-2 standard deviations below an international reference mean), weight-for-age Z-score (WAZ <-2 SD), and weight-for-height Z-score (WHZ <-2 SD) were used to define respectively stunting, underweight and wasting.
**Reply: Sentence was reworded**
144: data”were” . Data is a plural
**Reply: word was corrected**
Line 148: on comparing baseline characteristics of infants attending each visit to explore whether there was any alteration that could be imputed to the lost to follow-up.
Sentence is confusing. try instead “to explore whether any differences existed that could be associated with loss to follow up”
**Reply: Sentence was reworded, thank you**
Table 4: I’ve forgotten why Wasting is only assessed at month 1. I’d suggest the authors add a footnote to the table about that, because the authors obviously calculated prevalence of wasting at all times.

**Reply: there were no preterm infants with wasting at 9 and 12 months, that ist he reason why point estimates are not shown at these time-points.**

Reviewer #2: Most of my concerns have been addressed. However, it remains vague how the variables infant sex, maternal age and maternal literacy were selected for adjustments. What possible control variables did the authors considered at the beginning of the analysis? How did the end up in the three variables? This must be clearly described in the manuscript.

**Reply: This is a good and relevant point raised by the reviewer. Actually, more variables were measured for the mothers during their pregnancies, these included parity, trimester of first antenatal clinic visit, BMI and MUAC and infectious status. These parameters have been controlled in the analyses of the determinants of LBW.**

**For the current analysis of the effect of LBW on growth, nutrition and mortality, the maternal variables age and literacy were considered to potentially affect the way mothers provide care to their infants and therefore any differences in these parameters could be confounding the potential effect of LBW only. The infant sex has been taken as a forced variable as sex and age are mostly considered as counfounding factors.**

**A clarifying sentence has been added in the methods section.**
